# NOX2 Inhibition Impairs Early Muscle Gene Expression Induced by a Single Exercise Bout

**DOI:** 10.3389/fphys.2016.00282

**Published:** 2016-07-14

**Authors:** Carlos Henríquez-Olguín, Alexis Díaz-Vegas, Yildy Utreras-Mendoza, Cristian Campos, Manuel Arias-Calderón, Paola Llanos, Ariel Contreras-Ferrat, Alejandra Espinosa, Francisco Altamirano, Enrique Jaimovich, Denisse M. Valladares

**Affiliations:** ^1^Facultad de Medicina, Centro de Estudios Moleculares de la Célula, Instituto de Ciencias Biomédicas, Universidad de ChileSantiago, Chile; ^2^Laboratory of Exercise Sciences, Clínica MEDSSantiago, Chile; ^3^Facultad de Odontología, Institute for Research in Dental Sciences, Universidad de ChileSantiago, Chile; ^4^Facultad de Medicina, School of Medical Technology, Universidad de ChileSantiago, Chile

**Keywords:** redox signaling, antioxidant defense, reactive oxygen species, IL-6, NADPH oxidase, muscle adaptation

## Abstract

Reactive oxygen species (ROS) participate as signaling molecules in response to exercise in skeletal muscle. However, the source of ROS and the molecular mechanisms involved in these phenomena are still not completely understood. The aim of this work was to study the role of skeletal muscle NADPH oxidase isoform 2 (NOX2) in the molecular response to physical exercise in skeletal muscle. BALB/c mice, pre-treated with a NOX2 inhibitor, apocynin, (3 mg/kg) or vehicle for 3 days, were swim-exercised for 60 min. Phospho–p47^phox^ levels were significantly upregulated by exercise in *flexor digitorum brevis* (FDB). Moreover, exercise significantly increased NOX2 complex assembly (p47^phox^–gp91^phox^ interaction) demonstrated by both proximity ligation assay and co-immunoprecipitation. Exercise-induced NOX2 activation was completely inhibited by apocynin treatment. As expected, exercise increased the mRNA levels of manganese superoxide dismutase (MnSOD), glutathione peroxidase (GPx), citrate synthase (CS), mitochondrial transcription factor A (tfam) and interleukin-6 (IL-I6) in FDB muscles. Moreover, the apocynin treatment was associated to a reduced activation of p38 MAP kinase, ERK 1/2, and NF-κB signaling pathways after a single bout of exercise. Additionally, the increase in plasma IL-6 elicited by exercise was decreased in apocynin-treated mice compared with the exercised vehicle-group (*p* < 0.001). These results were corroborated using gp91-dstat in an *in vitro* exercise model. In conclusion, NOX2 inhibition by both apocynin and gp91dstat, alters the intracellular signaling to exercise and electrical stimuli in skeletal muscle, suggesting that NOX2 plays a critical role in molecular response to an acute exercise.

## Introduction

It is well known that regular exercise induces several beneficial effects in skeletal muscle, such as an increased insulin sensitivity (Kirwan et al., 2009), mitochondrial biogenesis (Hood et al., 2015), and endogenous antioxidant defense (Ristow et al., 2009). Multiple signaling pathways have been implicated in exercise-promoted skeletal muscle health, nonetheless the precise mechanisms involved in these effects are not fully understood (Neufer et al., 2015).

During the past decade, a large body of evidence demonstrated that reactive oxygen species (ROS) generation is upregulated during exercise in several tissues (Jackson, 2011). Exercise-induced ROS production acts as a signal to activate *redox* sensitive pathways that result in activation of transcription factors and gene expression, modulating both acute response and long-term training adaptation (Strobel et al., 2011). Moreover, training increases the expression of antioxidant enzymes as superoxide dismutase (SOD), glutathione peroxidase (GPx), and catalase (Ristow et al., 2009). It has been reported that ROS also participate in exercise-induced mitochondrial adaptation (Piantadosi and Suliman, 2012), such as increased expression and activity of citrate synthase (CS) (Strobel et al., 2011). Interestingly, ROS scavengers can impair early or long-term effects of exercise including antioxidant gene expression and mitochondrial biogenesis (Ristow et al., 2009; Petersen et al., 2012; Paulsen et al., 2014b; Qi et al., 2014).

The production of ROS has long been recognized as a critical component of skeletal muscle physiology, but the sites that generate superoxide and hydrogen peroxide during exercise have remained controversial (Jackson, 2016). Mitochondria have been traditionally considered as the major ROS source in skeletal muscle (Powers et al., 2011). However, recent studies have reported that during short periods of contraction, a rise in cytosolic ROS precedes and is greater than the increase in mitochondrial ROS generation (Pearson et al., 2014; Sakellariou et al., 2014), suggesting that non-mitochondrial ROS generation could be the major ROS source in skeletal muscle. Accordingly, xanthine oxidase (XO; Gomez-Cabrera et al., 2005) and NADPH oxidase (NOXs) emerge as possible candidates to participate in the *redox*-dependent skeletal muscle plasticity (Sakellariou et al., 2014) but the former appears not to have an important role in muscle adaptation (Wadley et al., 2013).

The NOXs are protein complexes that generate ROS in a highly regulated manner in response to cytokine, hormonal, and mechanical signals (Lambeth, 2004). NAPDH oxidase isoform 2 (NOX2) is expressed in skeletal muscles and it is composed of both catalytic and regulatory subunits (Espinosa et al., 2016). The gp91^phox^ and p22^phox^ subunits are localized mainly in the sarcolemma and transverse tubules, whereas the regulatory components: p47^phox^, p67^phox^, p40^phox^, and Rac1 are present in the cytoplasm (Hidalgo et al., 2006). Upon stimulation, the cytosolic subunits translocate to the membrane and full enzyme assembly takes place, accompanied by enzyme activation and ROS generation (Lambeth, 2004). The highly specific localizations and activation of NOX2 in skeletal muscle represent a high potential for spatial and temporal regulation of *redox* signaling in skeletal muscle physiology. However, the role of NOX2 in exercise-induced ROS signaling remains largely unknown.

The exercise-induced multi-systemic adaptation are mediated partially by the secretion of myokines from skeletal muscle (Pedersen and Febbraio, 2012). During the past years, IL-6 has emerged as an important cytokine to coordinate metabolic functions during and after exercise. This cytokine is produced and released into the bloodstream in response to muscle contraction (Keller et al., 2005). The molecular mechanism involved in exercise-induced IL-6 expression is still unclear, even though it has been suggested that ROS-regulated transcription factors participate in the signaling (Scheele et al., 2009). Supporting this hypothesis, we recently showed that electrical stimulation is able to increase IL-6 expression through activation of both NOX2 and of NF-κB transcriptional activity in skeletal muscle cells (Henríquez-Olguín et al., 2015).

The aim of this work was to study the contribution of NOX2 activation to intracellular signaling in response to a single bout of exercise in skeletal muscle. We hypothesize that exercise-induced NOX2 activation modulates early responses related with antioxidant and metabolic signaling in skeletal muscle.

## Methods

### Animal treatment

All animal procedures were in accordance with guidelines approved by the Bioethical Committee at the Facultad de Medicina, Universidad de Chile. Male 5–6 week-old BalbC mice (20–30 g) were intraperitoneally injected for 3 days with either the NOX2 inhibitor apocynin solution (3 mg/Kg) or vehicle as previously described (Khairallah et al., 2012). Apocynin (Sigma-Aldrich) solution was prepared in the dark at 12 mg/mL in absolute ethanol and then was diluted in sterile saline solution (0.9% NaCl) in a dosage that has shown previously to inhibit NOX2 *in vivo* (Khairallah et al., 2012). In another set of experiments, mice were injected for 2 days with Pyrrolidinedithiocarbamate (PDTC), a NF-κB inhibitor (50 mg/Kg). PDTC (Sigma-Aldrich) solution was prepared in sterile saline solution (0.9% NaCl) at 0.3 mg/mL for injections, as previously reported (Ji et al., 2004).

### Exercise protocol

Animals were divided into the following groups: Vehicle-treated sedentary, Vehicle-treated plus exercise, Apocynin-treated sedentary and Apocynin-treated plus exercise (*n* = 10 mice for each group). The swimming groups of mice were acclimated to swimming for 10 min per day for 5 days without weight in the tail in order to minimize potential stress response. Afterwards, the mouse with the indicated treatment was gently placed in the water and performed 60 min of swimming exercise with a weight (5% of their body weight) attached to the mouse-tail, this intensity has been proposed as moderate intensity in previous work and similar to other types of exercise (Contarteze et al., 2008). After the swimming time, mice were removed from the swimming pool, dried gently with paper towels and returned to their cages. A 1-liter beaker (11 cm diameter and 15 cm height) filled with water (32 ± 2⋅C) was used as a swimming pool to assess the exercise protocol. In another set of experiments mice were divided in two groups: Vehicle-treated and PDTC-treated plus exercise as described above (*n* = 5 mice for each group). For mRNA isolation, the animals were euthanized by cervical dislocation 2 h after the end of swimming exercise and *flexor digitorum brevis* (FDB) muscles were then stored at −80°C until needed (*n* = 5 mice per group). Plasma, total proteins and muscle cryosection samples were obtained immediately after the end of the exercise protocol (*n* = 5 mice per group).

### Adult skeletal muscle fiber isolation

FDB muscles were dissected from 5 to 6 week old mice as previously described (Altamirano et al., 2013). Briefly, muscle fibers were obtained by enzyme digestion of the whole muscle with collagenase type IV (2.7 mg/ml) (Worthington, Lakewood, NJ) for 90 min at 37°C followed by mechanical dissociation with fire polished Pasteur pipettes. Isolated fibers were seeded in ECM Gel-coated (Sigma-Aldrich) dishes in DMEM supplemented with 10% horse serum. After 20 h of seeding, the fibers were used for experimentation.

### Electrical stimulation (ES)

Isolated skeletal muscle fibers were stimulated with a stimulation device that consists of six rows of platinum wires, intercalated 0.5 cm apart, with alternate polarity across a circular Teflon holder that fits in the dish. This was connected to a Grass S48 pulse generator, as previously described (Valladares et al., 2013). The protocol for ES used was one train of 250 square wave pulses of 0.5 ms duration at a frequency of 20 Hz (12.5 s total stimulation time). This protocol was previously shown to be effective in inducing both Ca^2+^ signaling and gene expression in adult skeletal muscle fibers (Jorquera et al., 2013). For NOX2 inhibition, the fibers were incubated either with 50 μM apocynin (IC50 10 μM) or 5 μM of gp91-dstat (IC50 1 μM) 30 min before ES, these concentrations are able to inhibit NOX2 in adult muscle fibers (Díaz-Vegas et al., 2015) and skeletal myotubes (Henríquez-Olguín et al., 2015).

### Western blot

FDB muscles were homogenized using an electric homogenizer (Fluko, Shanghai, China) in a lysis buffer containing in mM: 20Tris-HCl (pH 7.5), 1% Triton X-100, 2 EDTA, 20 NaF, 1 Na_2_P_2_O_7_, 10% glycerol, 150 NaCl, 10 Na_3_VO_4_, 1 PMSF and protease inhibitors (Complete™, Roche Applied Science). Proteins were separated using SDS-PAGE and transferred to PVDF membranes. The following primary antibodies and their dilutions were used as follows: Total NF-κB total p65 subunit (1:1000; Cell Signaling); p-Ser536-p65 (1:1000; Cell Signaling); p47^phox^ (1:5000; Sigma); p-p47^phox^ (pSer359) (1:5000; Sigma); gp91^phox^ (1:2000; BD Transduction); p-p38 (thr180/tyr182) (1:2000; Santa Cruz); p38 (1:2000; Santa Cruz); Phospho-ERK1/2(Thr202/Tyr204)(1:2000; Cell Signaling); ERK 1/2 (1:2000; Santa Cruz); α-Tubulin (1:5000; Cell Signaling); anti-mouse IgG-HRP (1:20,000; Santa Cruz); anti-rabbit IgG-HRP (1:30,000; Thermo Scientific Pierce). The protein bands in the blots were visualized using a WESTAR Supernova detection kit (Cyanagen, Bologna, Italy) and ChemiDoc™ MP System (Bio-Rad, USA). The intensity of the bands was determined with ImageJ densitometry analysis.

### Co-immunoprecipitation assay and immunoblot (CoIP)

Total FDB muscle proteins were lysed for 1 h lysis buffer on ice (in mM: 20Tris-HCl pH 7.4, 0.1% Nonidet P-40, 5 EDTA pH 8, 10 EGTA pH 7.8, 140 NaCl, 10% glycerol and protease inhibitors). Whole muscle lysates (100 μg of protein) were spun at 15000 g for 20 min and the supernatant fraction was pre-cleared for 30 min with 10 μg of A/G agarose. Pre-cleared lysates were separated by centrifugation and then were incubated along with the immuno-precipitating antibody for 4 h. To pull down the immune-complexes we incubated the samples with 50 μg A/G agarose beads for 30 min. Beads were spun down by centrifugation and washed 3 times with washing buffer in mM (25 HEPES pH 7.5, 0.2% Nonidet P-40, 140 NaCl, 0.1% BSA, 10% glycerol and protease inhibitors). Proteins were resolved by SDS-PAGE in 7–10% gels, transferred to PVDF membranes and assayed with the corresponding antibodies.

### Proximity ligation assay (PLA)

Cryosections (10 μm thick) from adult mice FDB muscle were fixed using freshly prepared para-formaldehyde (4%) for 20 min, washed 3 times with 0.1 M PBS, pH 7.4, and blocked with PBS containing 5% BSA for 1h. P47^phox^/gp91^phox^ interaction was detected *in situ* using the Duolink® *In Situ* Red Starter Kit Mouse/Rabbit (Sigma-Aldrich) according to manufacturer instructions. Briefly, primary antibodies against p47^phox^ and gp91^phox^ were applied over night at 4°C in a humid chamber. Duolink plus and minus secondary antibodies against the primary antibodies were then incubated for 1 h at 37°C. These secondary antibodies were provided as conjugates to oligonucleotides that were tied together in a closed circle by Duolink Ligation Solution, provided that the antibodies were in close proximity (<40 nm). Finally, polymerase was added, to amplify any existing closed circles, and detection was achieved with complementary fluorescently labeled oligonucleotides. To define fiber structure, after PLA assay an immunofluorescence against dystrophin was performed.

### mRNA quantitation

Total RNA was isolated from FDB muscles from both the apocynin- or vehicle-treated groups with TRIzol® reagent (Invitrogen) according to the manufacturer's protocol. Additionally, the same extraction protocol was used to obtain total RNA from isolated fibers that were electrical stimulated. cDNA was prepared by reverse transcription (RT) reaction of 1 μg of total RNA using random primers. Real time PCR was performed as previously described (Altamirano et al., 2012) using the following primers: GAPDH: 5′-CTCATGACCACA GTCCATGC-3′ and 5′-TTCAGCTCTGGG ATGACCTT-3′, IL-6: 5′-CCAATTTCCAAT GCTCTCCT-3′ and 5′-ACCACAGTGAGG AATGTCCA-3′, CS: 5′-TGCTGGGGGTCT CCCTGTCC-3′ and 5′-TGGGACCAGGCC CGAAGAGG-3′, tfam: 5′-GCAAAGGATGAT TCGGCTCAGGGAA-3′ and 5′-CCGGATCGTTTC ACACTTCGACGG-3′, MnSOD: 5′-TGGCTTGGC TTCAATAAGGA-3′, and 5′-AAGGTAGTAAGC GTGCTCCCACAC-3′, Gpx: 5′-GGGCTCCCT GCGGGGCAAGGT-3′ and 5′-ATGTACTTG GGGTCGGTCATG-3′.

### IL-6 ELISA

IL-6 protein levels in plasma were quantified using a mouse IL-6 ELISA READY Kit (eBioscience) in each sample according to manufacturer's instructions. Mice were treated with the indicated inhibitor and then the plasma was collected immediately after 60 min of exercise through puncture. Absorbance was measured using a spectrophotometer (Synergy 2, BioTek, Winooski, USA).

### Statistical analysis

Data of n experiments were expressed as mean ± SD. The significance of difference among treatments was evaluated using a *t*-test for unpaired data or one-way ANOVA-followed by Tukey *post hoc*. A *P*-value < 0.05 was considered statistically significant.

## Results

### Exercise activates NOX2 in adult skeletal muscle

**Figure 1:**
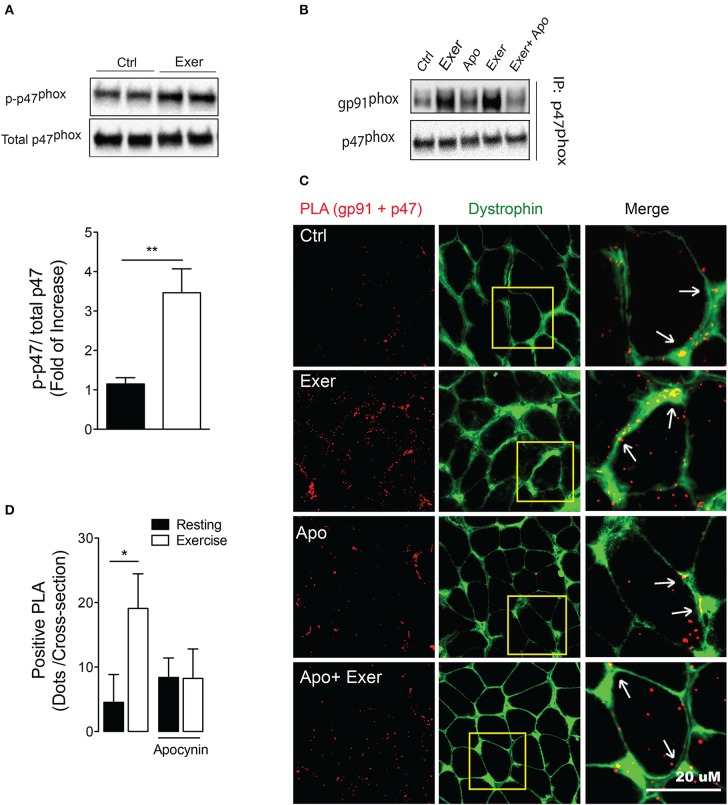
**Exercise-induced NOX2 activation was blocked by apocynin treatment. (A)** Representative blot and quantitation of phospho-p47^phox^ levels in FDB muscle under rest and exercise conditions. **(B)** gp91^phox^/p47^phox^interaction by co-immunoprecipitation in muscle lysates in control conditions (Ctrl), after exercise (Exer), control with apocynin (Apo, NOX2 blocker) and exercise without and with apocynin. **(C)** Representative *in situ* proximity ligation assay (PLA) probes and **(D)** quantification for gp91^phox^ and p47^phox^ interaction as PLA dots after exercise in control and apocynin-treated mice. Anti-dystrophin antibody was used to define fiber perimeter. Arrows show colocalization of the PLA signal (red) with dystrophin (green). Data are expressed as mean ± SD from five different determinations. *p < 0.05, **p < 0.01; one-way ANOVA-*Tukey* and *t*-*student* were applied.

Recent studies have shown that contractions are accompanied by a large cytosolic ROS production (Sakellariou et al., 2013; Pearson et al., 2014), and suggest a major role for non-mitochondrial sources (Sakellariou et al., 2014). To address whether an acute endurance exercise can activate NOX2 in skeletal muscle, mice performed 60 min of non-exhaustive swimming exercise. We studied p47^phox^ phosphorylation immediately following exercise. Swimming exercise induced significant increases of phospho-p47^phox^ levels compared with unexercised mice (~3-fold, *p* < 0.01; Figure 1A).

To address whether apocynin is blocking the NOX2 assembly, we determined interactions between cytosolic (p47^phox^) and membrane-bound subunit (gp91^phox^) in FDB muscle using two approaches. First, we showed that swimming exercise increases the protein-protein interaction between these subunits measured by co-immunoprecipitation. More importantly, the interaction between p47^phox^ and gp91^phox^ was disrupted by apocynin, a NOX2 blocker, treatment (Figure 1B). Second, we used the proximity ligation assay (PLA) to further demonstrate interactions between NOX2 subunits. This technique shows proximity as fluorescence dots when the two proteins assessed are nearer than 40 nm. Swimming exercise increased around 4-fold the number of positive PLA dots (p47^phox^–gp91^phox^ proximity) in FDB muscle (*p* < 0.05; Figures 1C,D). Interestingly, exercise-induced p47^phox^–gp91^phox^ proximity was prevented by apocynin treatment, without any effect in the basal levels (Figures 1C,D).

### NOX2 activation is necessary for ERK1/2, p38, and NF-κb signaling induced by exercise

**Figure 2:**
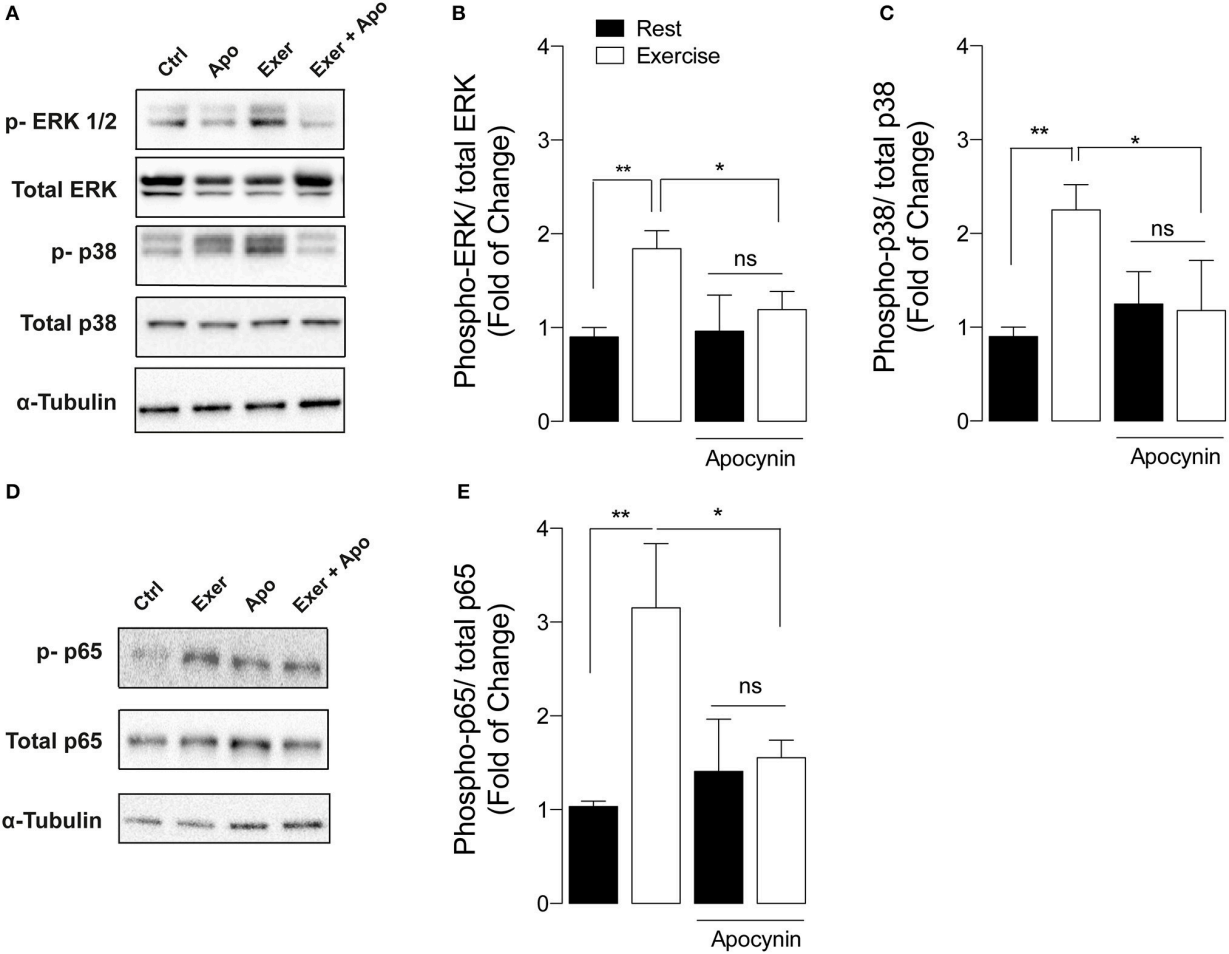
**NOX2 inhibition reduces exercise-induced MAPK and NF-κB activation in skeletal muscle**. **(A)** Representative and **(B,C)** quantification of western blot showing ERK ½, and p38 phosphorylation after exercise in both apocynin and vehicle-treated mice. **(D)** Representative blot and **(E)** quantification of p-p65 NF-κB levels from both apocynin and vehicle-treated mice. Data are expressed as mean ± SD from five different determinations. *p < 0.05, **p < 0.01; and ns, no significant difference, one-way ANOVA-*Tukey.*

General ROS scavengers reduce the mitogen-activated protein kinase (MAPK) phosphorylation during exercise (Paulsen et al., 2014a). In order to determine the role of NOX2 in exercise-induced MAPK activation, we studied the changes of these pathways by Western blot using phospho-specific antibodies. Swimming exercise significantly increased ERK ½ (Thr202, Tyr204) and p38 phosphorylation by ~2-fold (*p* < 0.01), compared with unexercised mice in FDB (Figures 2A,C) and Gastrocnemius (data not shown) muscles. Interestingly, apocynin treatment totally blocked these activations in FDB muscle from exercised mice (Figures 2B,C).

NF-κB is a classic *redox* sensitive transcription factor that controls the expression of antioxidant enzymes and the myokine IL-6 (Ji et al., 2004). To determine if exercise-induced NOX2 activation participates in NF-κB signaling pathway activation, we measured p-p65 levels after exercise in vehicle- and apocynin-treated mice. Swimming increased p65 phosphorylation (Ser536) by 3-fold in FDB muscle compared with control mice (Figures 2D,E, *p* < 0.01). In addition, apocynin treatment impaired NF-κB activation in exercised muscle (Figures 2D,E, *p* < 0.05).

### Exercise-induced muscle and plasma IL-6 increments were blunted by NOX2 inhibition

**Figure 3:**
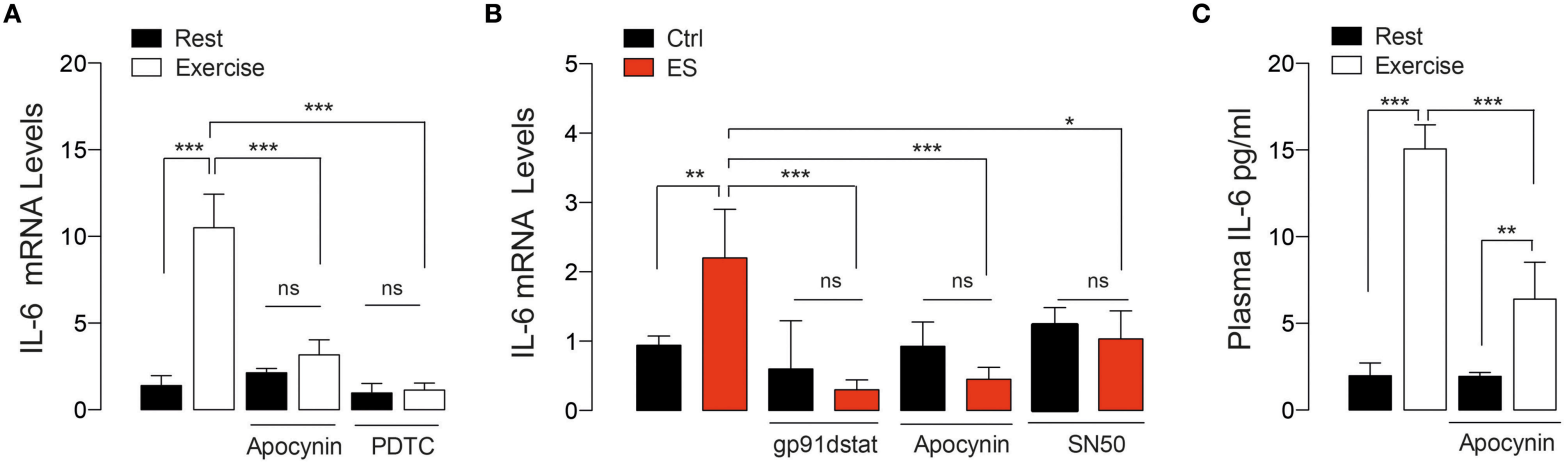
**NOX2-dependent ROS production is involved in IL-6 gene expression induced by both *in vivo* and *in vitro* exercise**. **(A)** Levels of muscle IL-6 mRNA after exercise in mice treated with vehicle, apocynin and PDTC (NF-κB inhibitor). **(B)** Levels of IL-6 mRNA in isolated skeletal muscle fibers that were pre-incubated with either apocynin or gp91-dstat (NOX2 blockers) or SN50 (NF-κB inhibitor) before electrical stimulation (ES). **(C)** Plasma IL-6 levels obtained immediately after exercise in both apocynin- or saline-treated mice. Data are expressed as mean ± SD from five different determinations. *p < 0.05, **p < 0.01, ***p < 0.001; ns, no significant difference, one-way ANOVA-*Tukey.*

IL-6 is a key myokine involved in acute and chronic response to exercise in skeletal muscle (Pedersen and Febbraio, 2012). In order to study the participation of NOX2-activation on IL-6 expression, we measured IL-6 mRNA content in swimming exercised FDB muscle. Exercise induced a robust increment in IL-6 mRNA by ~7.5-fold compared to control mice (*p* < 0.001), which was blocked by apocynin (Figure 3A). In order to determine whether the apocynin effect was due to NOX2 blockage, we corroborated these results using the peptide gp91-dstat, a specific NOX2 inhibitor, on isolated FDB fibers in response to electrical stimulus (ES, an *in vitro* exercise protocol) (Figure 3B). In isolated FDB fibers, IL-6 mRNA levels were upregulated by ES and this increase was blocked by NOX2 inhibitors apocynin (50 μM) and gp91-dstat (5 μM). Additionally, we evaluated the participation of NF-κB in exercise induced IL-6 expression. When exercised mice were treated with PDTC (a NF-κB inhibitor) the increase in IL-6 expression was totally blunted (Figure 3A). Similar result was obtained in isolated fibers incubated with the NF-κB inhibitor SN50 (10 μM) and electrically stimulated (Figure 3B).

We also evaluated the plasma IL-6 concentration immediately after exercise in vehicle- and apocynin-treated mice. Figure 3C shows that plasma IL-6 was increased ~7-fold in exercised-mice compared to control (1.9 vs. 14.7 pg/ml, *p* < 0.001). The apocynin–treated group showed ~40% less plasma IL-6 increase compared to vehicle-treated in exercising conditions (6.4 vs. 14.7 pg/ml, *p* < 0.01).

### NOX2-activation is required for exercise-induced antioxidant and mitochondrial mRNA markers

**Figure 4:**
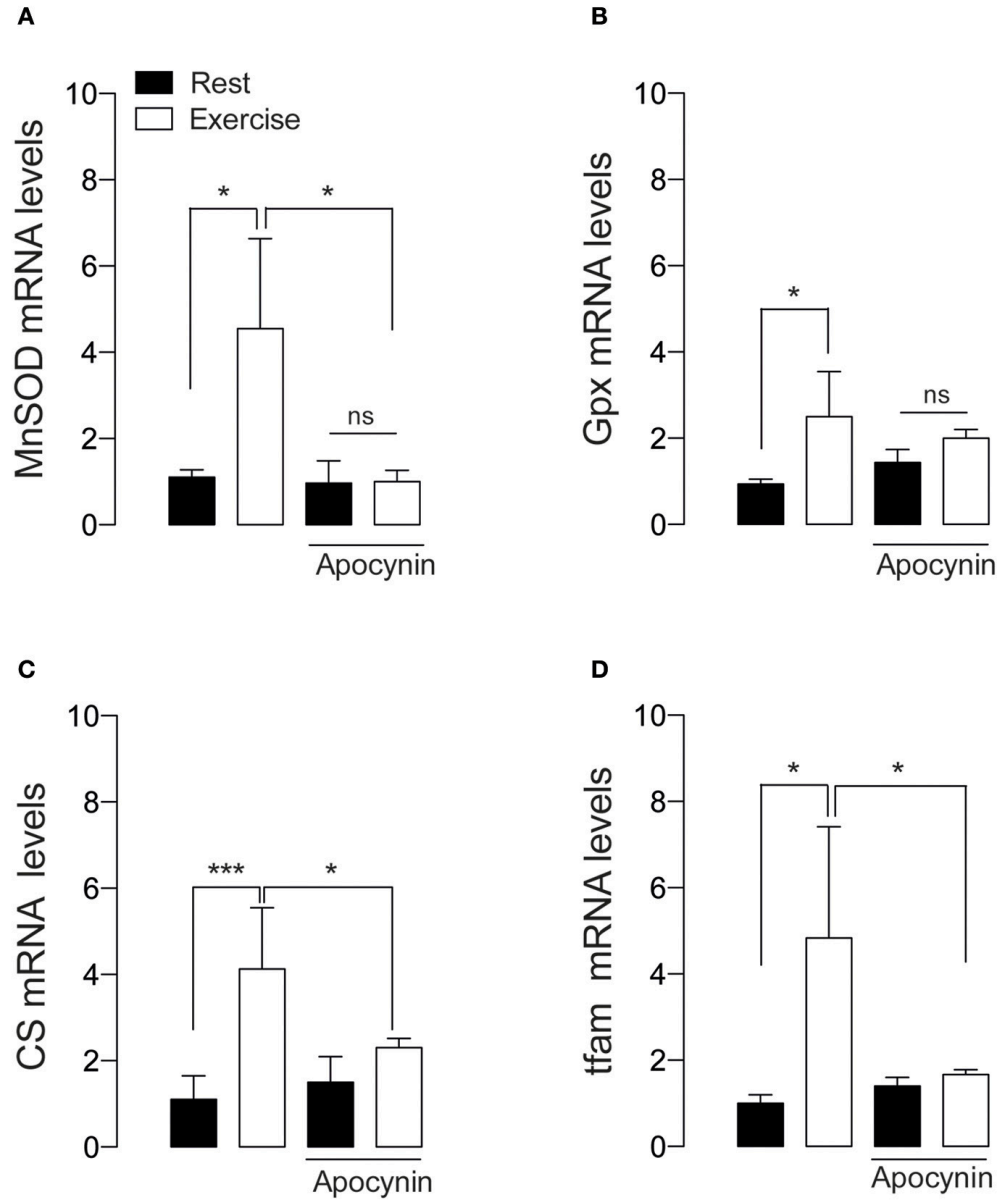
**NOX2 activation is necessary for exercise-induced antioxidant and mitochondrial gene expression**. **(A)** Levels of mRNA for manganese superoxide dismutase (MnSOD), **(B)** Glutathione peroxidase (Gpx), **(C)** Citrate synthase (CS), and **(D)** mitochondrial transcription factor A (tfam) mRNAs were assessed by real time PCR in FDB muscle after swimming exercise. Data are expressed as mean ± SD from five different determinations. *p < 0.05, ***p < 0.001; ns, no significant difference, one-way ANOVA-*Tukey.*

We studied the changes in antioxidant and metabolic mRNA markers in FDB muscle lysates 2 h after 60 min of swimming exercise using real time RT-qPCR. Swimming exercise significantly increased the mRNA levels of manganese superoxide dismutase (MnSOD) (Figure 4A), and Glutathione peroxidase (GPx) (Figure 4B) in 2.5- and 4.5-fold, respectively (*p* < 0.05). Interestingly, these effects were partially ablated by apocynin treatment (Figures 4A,B).

Tfam is necessary and sufficient to induce mitochondrial biogenesis (Ikeuchi et al., 2005). We addressed whether NOX2 inhibition can impair mitochondrial biogenesis signaling by studying the changes in the mRNA levels of mitochondrial transcription factor A (tfam) and citrate synthase (CS). Our results show that the levels of both tfam and CS mRNA were increased about 4 folds under exercising conditions (*p* < 0.05 and *p* < 0.01 respectively, Figures 4C,D). Apocynin treatment prevented the exercise-induced mRNA increment, suggesting the participation of NOX2 in exercise-induced mitochondrial gene activation in skeletal muscle.

**Figure 5:**
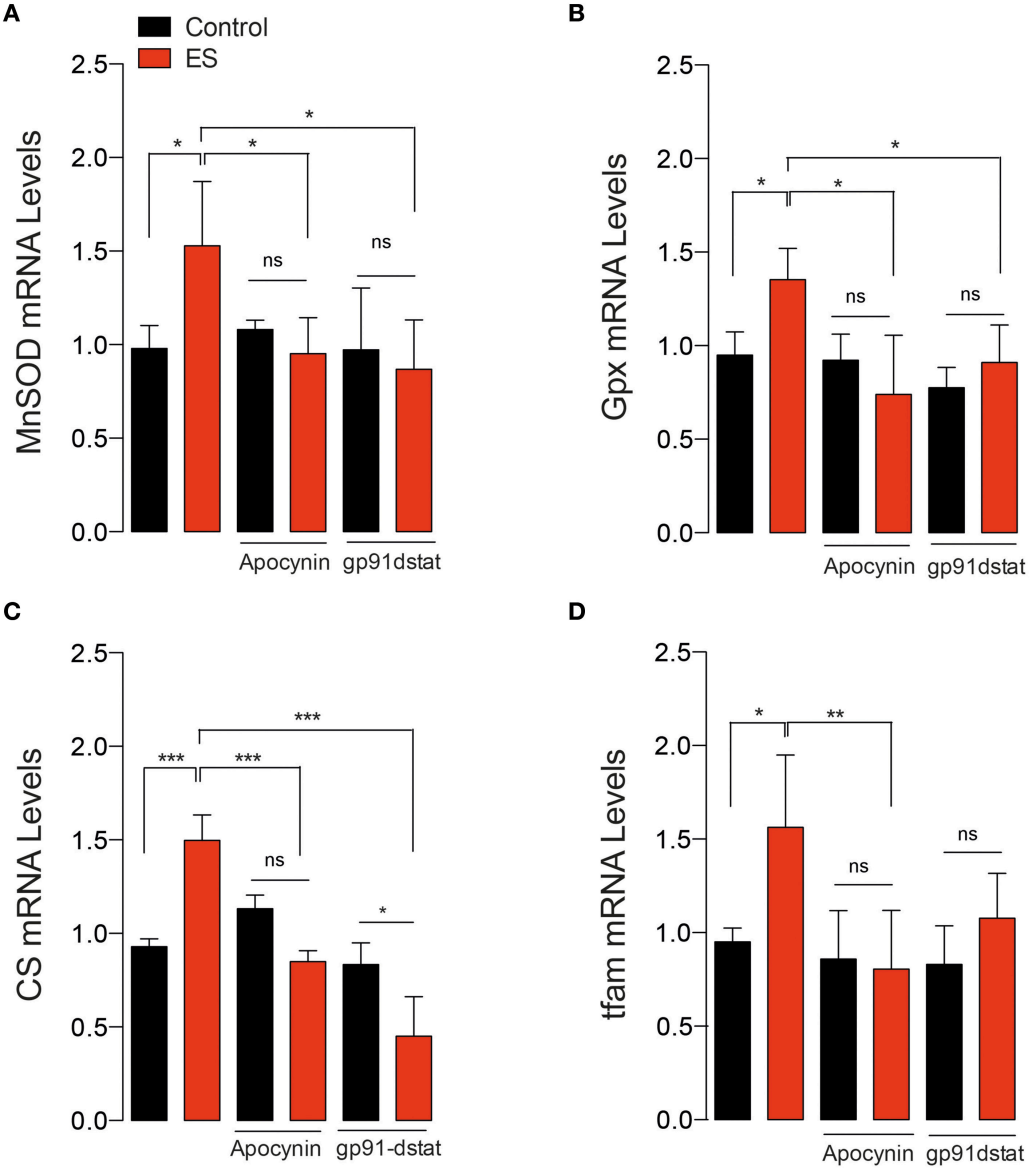
***In vitro* electrical stimulation-induced gene expression in a NOX2-dependent manner**. **(A)** Levels of mRNA for manganese superoxide dismutase (MnSOD), **(B)** Glutathione peroxidase (Gpx), **(C)** Citrate synthase (CS) and **(D)** Mitochondrial transcription factor A (tfam). Gene transcription was assessed by real time PCR in isolated skeletal muscle fibers in the presence and absence of the NOX2 inhibitor gp91-dstat before electrical stimulation (ES). Data are expressed as mean ± SD from five different determinations. *p < 0.05, **p < 0.01, ***p < 0.001; ns, no significant difference, one-way ANOVA-*Tukey.*

We also corroborated these results using the peptide gp91-dstat on isolated FDB fibers in response to electrical stimulation. As shown in Figure 5, ES significantly increased mRNA levels of MnSOD, GPx, CS and tfam. Both apocynin (50 μM) and gp91-dstat (5 μM) completely blunted the increase in mRNA levels induced by ES for all the studied mRNAs.

## Discussion

The present study aims to determine the participation of NOX2 in molecular events induced by a single bout of exercise in skeletal muscle. The main finding was that endurance exercise activates NOX2, and subsequently modulates ROS-sensitive pathways to promote gene transcription. To the best of our knowledge, this is the first study to show *in vivo* NOX2 activation during exercise and its participation in exercise–induced *redox* signaling in skeletal muscle.

ROS act as signaling molecules that participate in training-induced skeletal muscle adaptation (Gomez-Cabrera et al., 2008; Ristow et al., 2009; Strobel et al., 2011). There is increasing evidence that localized ROS signaling at specific subcellular compartment is essential for *redox* sensitive pathways modulation (Ushio-Fukai, 2006). Thus, the ROS source that contributes to exercise-induced molecular signaling could be a key piece of skeletal muscle physiology (Jackson et al., 2016 for review). It has been previously reported that xanthine oxidase-derived ROS is a dispensable source for skeletal muscle adaptation following 6-weeks of endurance training (Wadley et al., 2013). More recently, several groups have shown that NOX2 is the major ROS source during muscle contraction in both cultured myotubes and isolated fibers (Espinosa et al., 2013; Sakellariou et al., 2013; Pearson et al., 2014; Díaz-Vegas et al., 2015; Henríquez-Olguín et al., 2015). However, the experimental evidence showing that exercise is sufficient to activate NOX2 was still lacking. In the present study, we showed that an acute endurance exercise was a powerful stimulus for NOX2 activation in FDB muscle. The fact that exercise induces NOX2 activation has meaningful implications for understanding the role of localized *redox* homeostasis in striated muscle (Sánchez et al., 2008; Hidalgo and Donoso, 2011; Wang et al., 2015). We propose that NOX2 plays a critical role in regulated ROS-dependent signaling in skeletal muscle induced by endurance exercise. Even though the evidence support that ROS generated by NOX2 participate in signal transduction, the mechanisms by which contraction activates NADPH oxidase and regulates ROS generation are poorly understood. Recently, our laboratory has proposed that ATP release from skeletal muscles can act as NOX2 activator through activation of P2Y_1_ receptors and a PKC-dependent pathway (Díaz-Vegas et al., 2015). Future works should determine the mechanisms involved in *in vivo* exercise-induced NOX2 activation.

Apocynin has been widely used as a NOX2 inhibitor (Sánchez et al., 2008; Yokota et al., 2009; Khairallah et al., 2012; Espinosa et al., 2013; Díaz-Vegas et al., 2015; Henríquez-Olguín et al., 2015), however its use is controversial. Some *in vitro* studies have shown that apocynin can induce ROS generation or has antioxidants properties (Vejrazka et al., 2005). However, the efficacy of apocynin during *in vivo* conditions has largely been reported (Khairallah et al., 2012; Espinosa et al., 2013; Yamacita-Borin et al., 2015; Cheng et al., 2016; Zhu et al., 2016). In the present study, we confirmed that apocynin is able to disrupt the assembly of the NOX2 complex during *in vivo* exercise. The results obtained *in vitro* using both apocynin and gp91-dstat peptide confirm that NOX2 inhibition alter intracellular signaling induced by exercise and electrical stimulation. Even though the evidence support that apocynin is blocking NOX2 assembly and ROS production, we cannot exclude other side effects of our treatment. Future studies should explore gene silencing and genetic tools for NOX2 inhibition.

It is well described that exercise activates ERK and p38 MAPK pathways in animal (Goodyear et al., 1996) and human (Osman et al., 2000) skeletal muscle in a *redox* sensitive manner (Paulsen et al., 2014a). During contraction, activation of the p38 MAPK in adult skeletal muscle results in enhanced mitochondrial biogenesis (Akimoto et al., 2005). Different research groups have shown that ROS scavengers attenuate p38 and ERK activation induced by exercise in muscle tissue (Michailidis et al., 2013). In human skeletal muscle precursor cells, NOX2 has proven to be critical for both myoblast proliferation and differentiation through ERK 1/2 pathway (Mofarrahi et al., 2008). Interestingly, the present study showed that NOX2 inhibition impairs MAPK activation after a single bout of endurance exercise in skeletal muscle, suggesting that ROS-derived from NOX2 are necessary for this pathways activation.

NF-κB is a well-described *redox* sensitive transcription factor (Michailidis et al., 2013; Wadley et al., 2013), which is activated by exercise in skeletal muscle (Aoi et al., 2004; Ji et al., 2004). It has been proposed that exercise-induced NF-κB activation participates in acute and chronic responses to exercise (Feng et al., 2013). For example, PDTC administration reduces exercise-induced antioxidant gene expression (Ji et al., 2004) and impairs mitochondrial biogenesis after long-term training (Feng et al., 2013), suggesting that NF-κB is essential for adequate response to exercise/training. It is well described that NOX2 induces NF-κB activation in several tissues (Brar et al., 2002; Yao et al., 2007; Li et al., 2009) including skeletal muscle cells (Mofarrahi et al., 2008). We have previously shown that NOX2 is required for transcriptional activation of NF-κB induced by electrical stimulation in skeletal myotubes (Henríquez-Olguín et al., 2015). Here, our results demonstrate that exercise-induced phospho-p65 NF-κB increment was blunted in apocynin-treated mice. Together, these antecedents suggest that transcriptional activation of NF-κB could be modulated by NOX2-dependent ROS production during exercise in skeletal muscle.

Myokines play a critical role modulating whole body metabolism during endurance and resistance exercise (Steensberg et al., 2000; Glund et al., 2007). A close relationship between ROS production and myokine production during exercise has been suggested (for review see Scheele et al., 2009). For example, IL-6 is a well-established myokine released by the skeletal muscle during exercise, whose levels are decreased by general antioxidants supplementation (Fischer et al., 2004; Yfanti et al., 2012). Moreover, in adipose tissue, NOX2 deficient mice have reduced IL-6 expression (Costford et al., 2014). Interestingly, in the present work, swimming and running (data not shown) increased both plasma and muscle mRNA of IL-6 in vehicle-treated mice; these effects were decreased by apocynin treatment. Additionally, both apocynin and gp91dstat decreased electrical stimuli-induced IL-6 gene expression in single fibers. On the other hand, unpublished observations from our lab using a mitochondrial-target antioxidant EUK-134, was not able to disrupt electrical stimulation-induced IL-6 expression. Together, these data strongly suggest NOX2-derived ROS production is critical for IL-6 expression and the subsequent release from skeletal muscles during exercise.

IL-6 gene has binding sites for NF-κB in its promoter regions (Libermann and Baltimore, 1990) and also we described that IL-6 expression induced by electrical stimulation is regulated by NF-κB activity in skeletal myotubes (Juretić et al., 2006; Henríquez-Olguín et al., 2015). Here, the results show that IL-6 expression following exercise or electrical stimulation is lowered by both PDTC and SN50, known NF-κB inhibitors. Together, these data demonstrated that NOX2 plays a critical role in NF-κB activation and the subsequent modulation of IL-6 gene expression in skeletal muscle under exercising conditions.

It is well known that antioxidant gene expression is increased in response to exercise training in mice (Hollander et al., 2001) and humans (Ristow et al., 2009). Our results show that skeletal muscle responds to a single bout of exercise or electrical stimulus with an increase in the transcription of both MnSOD and GPx genes. Moreover, NOX2 inhibition, by apocynin or gp91-dstat, prevents the exercise-induced mRNA upregulation. Our findings are in agreement with previous studies showing that general ROS scavengers decrease training/exercise-induction of both MnSOD and GPx gene expression in animal (Gomez-Cabrera et al., 2008) and human (Ristow et al., 2009; Petersen et al., 2012) skeletal muscle. Interestingly, MnSOD and GPx present NF-κB-binding sites in their promoter regions (Allen and Tresini, 2000), suggesting a NOX2/NF-κB pathway that may be involved in the regulation of gene activation induced by exercise.

Mitochondrial biogenesis is a key adaptation to increase endurance capacity (Hood, 2009). Tfam is a transcription factor necessary and sufficient for mitochondria biogenesis through the upregulation expression of mitochondrial proteins and other factors involved in mtDNA transcription and replication (Ikeuchi et al., 2005). The present study provides evidence that NOX2 inhibition blunts the Tfam mRNA upregulation induced by both exercise and electrical stimulation. This result is in line with previous studies showing that ROS scavengers as vitamin C disrupt training-induced tfam mRNA increment in rat skeletal muscle (Gomez-Cabrera et al., 2008). Citrate synthase (CS) has been extensively used as a metabolic marker for assessing mitochondrial oxidative capacity after endurance and resistance training (Larsen et al., 2012). NOX2 inhibition by both gp91dstat and apocynin reduces the CS mRNA after exercise or ES. These results may explain previous reports where dietary antioxidants reduced mitochondrial content in response to exercise (Gomez-Cabrera et al., 2008; Ristow et al., 2009; Strobel et al., 2011; Paulsen et al., 2014a). Our data support the hypothesis that exercise-induced mitochondrial gene expression is a *redox* sensitive process (Sano and Fukuda, 2008) and it is affected by specific inhibitors of NOX2-dependent ROS production. Adaptations to exercise training is produced from the cumulative effect of transient increases in mRNA transcripts that encode for various proteins after each successive exercise bout (Perry et al., 2010). Here, we show that an acute exercise is a powerful stimulus to activate NOX2 and promote adaptive molecular response to exercise in skeletal muscle. However, we cannot directly extrapolate our results to long-term training adaptation.

In summary, we provide evidence indicating that NOX2 is activated by endurance exercise promoting downstream pathways and gene activation in skeletal muscle. These findings provide insights into our understanding ROS homeostasis and ROS signaling induced by exercise in skeletal muscle. The role of NOXs in metabolic adaptation and long-term training effects remains to be determined.

## Author contributions

The study concept was developed by CH, AD, and DV. All authors were involved in executing the experiments, data collection and conducting measurements. CH, EJ, and DV, performed data analysis and were responsible for writing the manuscript. All authors contributed to the intellectual content and editing of the manuscript, and approved the final version.

## Funding

FONDECYT 1151293 (EJ), FONDECYT 3140491 (DV) and PhD Fellowships (CH, ADV, YUM and MAC) funded this work by Comisión Nacional de Ciencia y Tecnología (CONICYT).

### Conflict of interest statement

The authors declare that the research was conducted in the absence of any commercial or financial relationships that could be construed as a potential conflict of interest.
